# Hidradenitis suppurativa patient requiring cardiac procedure with inguinal access: Case management with ertapenem

**DOI:** 10.1177/2050313X241274819

**Published:** 2024-08-19

**Authors:** Laurence Mainville, Jimmy MacHaalany, Hélène Veillette

**Affiliations:** 1 Division of Dermatology, Department of Medicine, CHU de Québec-Université Laval, Québec, QC, Canada; 2 Division of Cardiology, Department of Medicine, CHU de Québec-Université Laval, Québec, QC, Canada; 3 Axe maladies infectieuses et immunitaires, Centre de Recherche du Centre Hospitalier Universitaire de Québec–Université Laval, Québec, QC, Canada

**Keywords:** Hidradenitis suppurativa, ertapenem, cardiac catheterization

## Abstract

Hidradenitis suppurativa is a chronic inflammatory skin disease that usually presents in young adults with painful abscesses in intertriginous areas. We present a case of severe hidradenitis suppurativa (Hidradenitis Suppurativa Investigator Global Assessment (HS-IGA) = 5; Hurley stage III) investigated by cardiology and respirology specialists for dyspnea. The patient’s symptoms required right-sided cardiac catheterization via the right femoral vein in the inguinal area. The patient was able to undergo this invasive cardiac procedure without infectious complications using multidisciplinary management (dermatology, cardiology, respirology, internal medicine, and infectious diseases specialists), intravenous ertapenem 1 g/day for 6 weeks perioperatively, biologic therapy, and treatment of diabetes with semaglutide. The administration of ertapenem preoperatively and postoperatively of an invasive procedure can be beneficial, particularly when the upcoming intervention requires access to skin areas severely affected by hidradenitis suppurativa. Comorbidities such as obesity and diabetes should be addressed as their treatment might contribute to improve hidradenitis suppurativa.

## Introduction

Hidradenitis suppurativa (HS) is a chronic inflammatory skin disease that usually presents in young adults with painful abscesses in intertriginous areas. Therapeutic options for HS include systemic antibiotics, biologics, and surgery.^1^ In this case report, we present an unexplored therapeutic indication with ertapenem for the treatment of severe HS awaiting invasive procedure.

## Case report

A 59-year-old woman was referred to dermatology by her cardiologist for skin lesions in the inguinal areas. Her medical history included dyslipidemia, leg edema, sleep apnea, and morbid obesity (body mass index (BMI) = 60). Her medication consisted of aspirin, atorvastatin, and furosemide. She had no allergy and was nonsmoker. She was being investigated by cardiology and respirology for dyspnea. She had undergone coronary angiography using a right radial approach, but the findings could not explain her dyspnea. Right heart cardiac catheterization was therefore necessary to complete the investigation, which required access via the right femoral vein. The cardiologist who examined the patient noted numerous skin lesions with abundant purulent discharge in her inguinal areas, which made venous access impossible to perform. The patient was sent to dermatology for evaluation and treatment before pursuing her workup.

The patient presented nodules and abscesses in the inguinal, pubic, axillary, and infra-mammary regions since puberty. Symptoms had been worsening over the last 2 years.

On physical examination, she had 5 nodules, 0 abscesses, and >15 draining fistulas in the inguinal regions (HS Investigator Global Assessment (HS-IGA) = 5), axillary comedones, and scars. A new diagnosis of severe HS (Hurley stage III) was made.

The patient was started on minocycline 100 mg/day for 3 months, and metformin 500 mg thrice per day for 2 months. She experienced diarrhea as a side effect, with only minor skin improvement. A pre-biologic workup was conducted, including a negative interferon-gamma release assay, as well as negative serologies for HIV and viral hepatitis. Elevated glycated hemoglobin (HbA1c 5.8%) indicated prediabetes. Doxycycline 100 mg/day was introduced for 2 months as a bridging regimen toward adalimumab. Adalimumab was initiated using 160 mg subcutaneously (s/c) at day 0; 80 mg s/c at day 14 (standard loading doses in HS); and 80 mg s/c weekly from day 28. The decision to use a dosage of 80 mg s/c weekly was supported by data indicating that adalimumab efficacity is inversely correlated with BMI.^2^ The patient was referred to internal medicine to treat her newly diagnosed diabetes, and she was started on semaglutide. At week 7 of adalimumab, symptoms of discharge in the inguinal areas had improved by 50%. Meanwhile, the patient also lost 10 kg under semaglutide.

In preparation for right-sided cardiac catheterization, the patient was managed in close collaboration with cardiology, dermatology, and infectious diseases specialists. She received intravenous ertapenem 1 g/day for 4 weeks. Complete blood counts and renal function were normal before treatment. C-reactive protein was elevated to 66.7 mg/L (normal range <10.0 mg/L). After week 4 with ertapenem, discharge in the inguinal areas had further improved. Cardiac catheterization using right femoral venous access was performed without any complication. Findings showed mild pulmonary hypertension and her dyspnea was thought to be likely due to her obesity and deconditioning. Intravenous ertapenem 1 g/day was continued for 2 weeks post cardiac catheterization, for a total treatment duration of 6 weeks. Enteral trimethoprim-sulfamethoxazole thrice weekly, topical metronidazole, and topical ciclopirox olamine were added after the end of intravenous antibiotics. Adalimumab 80 mg s/c weekly was continued to prevent HS relapses. The patient’s skin lesions remained stable over the following months.

## Discussion

Bacterial skin infection is not a frequent comorbidity in HS patients; screening is not routinely recommended by the US and Canadian Hidradenitis Suppurativa Foundations, nor by European guidelines.^3,4^ A negative swab, or normal skin microbiota, is even suggestive of HS.^4^

Nonetheless, local and systemic antibiotics are often used to treat HS flares, pointing toward the potential implication of microbial alterations in the disease course.^1^ There is evidence of skin dysbiosis in HS; anaerobic bacteria (*Porphyromonas* and *Peptoniphilus* species) and coagulase-negative staphylococci species are more abundant in lesional HS skin than non-lesional HS skin or healthy patient skin.^5,6^ Interestingly, Lee et al. investigated the association of HS with 45 infections reported in an American national registry. Using multivariate regression analysis, an association was shown between HS and “any serious infection” (adjusted odds ratio (aOR) 2.295; 95% confidence interval (CI) 2.208–2.385; *p* < 0.0001), and with septicemia (aOR 1.568; 95% CI 1.452–1.693; *p* < 0.0001).^7^ However, the design of this retrospective epidemiological study does not allow to conclude on a causal association between HS and the reported infections.

The management of HS with intravenous ertapenem was initially reported by Join-Lambert et al. in a retrospective study (*n* = 30). Patients’ median Sartorius score improved by 60% after ertapenem (6 weeks). Consolidation treatments were required after ertapenem to prevent HS relapses.^8^ In a retrospective medical chart review, American authors reported improvement of HS in 35/36 (97.2%) patients following treatment with ertapenem.^9^ On the basis of these studies, daily intravenous ertapenem for 6 weeks is now recommended as a third-line therapy, either as a bridge to dermatologic surgery or in context of rescue therapy.^1^ A recent study indicates that ertapenem can be administered for 12–16 weeks.^10^

The administration of ertapenem preoperatively and postoperatively of an invasive procedure can be beneficial, particularly when the upcoming intervention requires access to skin areas severely affected by HS. The patient described in this article suffered from uncontrolled severe HS. Her skin disease had the consequence to postpone an important cardiac procedure requiring access to major vessels in the groin. In addition to a long-term treatment such as biologic therapy, intravenous ertapenem administered perioperatively during 6 weeks can be a safe and effective therapeutic option for HS patients requiring rapid control of their disease. Incidentally, the proposed treatment can reassure clinicians who fear infectious complications while performing invasive surgical procedures in sites of severe HS lesions.

Finally, comorbidities such as obesity and diabetes should be addressed as their treatment might contribute to improve HS. Referral to internal medicine and semaglutide can be beneficial options.
